# Recent Advances in Thyroid Cancer Research

**DOI:** 10.3390/ijms23094631

**Published:** 2022-04-22

**Authors:** Daniela Grimm

**Affiliations:** 1 Department of Biomedicine, Aarhus University, Ole Worms Allé 4, 8000 Aarhus, Denmark; dgg@biomed.au.dk; Tel.: +45-21379702; Fax: +45-8612-8804; 2 Department of Microgravity and Translational Regenerative Medicine, University Clinic for Plastic, Aesthetic and Hand Surgery, Otto von Guericke University, Pfälzer Str. 2, 39106 Magdeburg, Germany; 3 Research Group “Magdeburger Arbeitsgemeinschaft für Forschung unter Raumfahrt-und Schwerelosigkeitsbedingungen” (MARS), Otto von Guericke University, 39106 Magdeburg, Germany

This Special Issue (SI) “*Recent Advances in Thyroid Cancer Research*” covers research articles and reviews in the field of thyroid cancer research. It includes publications reporting the results of cell biological studies, animal investigations and clinical studies.

The thyroid or *glandula thyroidea* is an endocrine gland located in the neck below the larynx in front of the trachea in mammals. In humans, it has the shape of a butterfly. It consists of two lobes connected by a narrow bridge called the isthmus. Thyroid carcinoma or thyroid cancer (TC) is a malignant tumor of the thyroid gland. It is the most common malignancy of the endocrine (hormonal) system, but is very rare, accounting for about 1% of all malignant tumors, however, it has been increasing in frequency in recent years.

The latest Global Cancer Observatory survey from 2020 reports that TC is responsible for 586,000 cancer cases worldwide [1]. The American Cancer Society published this year that there will be about 43,800 new cases of TC (11,860 in men and 31,940 in women) and about 2230 deaths from this type of tumour (1070 men and 1160 women) in the United States in 2022 [2].

TC is a malignant tumor of the glandular tissue of the thyroid gland. There are different types of thyroid cancer that arise from different cells in the thyroid gland. TC comprises several categories: (1) differentiated TC (DTC), including papillary (PTC), follicular (FTC), and Huerthle cell tumours, (2) medullary TC (MTC) and (3) anaplastic TC. The long-term survival rate of patients with differentiated TC is about 90%, whereas patients with poorly differentiated TC types show a long-term survival rate of below 10% due to their resistance to standard treatment options [3,4].

Standard therapy strategies for advanced and radioiodine-refractory TC are immunotherapy, chemotherapy and kinase inhibitors [5]. Unfortunately, the survival rate is still low, and therefore new research approaches with novel technologies are necessary.

This SI published five research articles [6,7,8,9,10]. Four studies investigated different types of TC cells (TCC) [6,7,9,10] and in three publications the cell cultures were combined with an animal study as tumor model [6,9,10]. Furthermore, one research article focused on PTC in a large South Eastern cohort of patients [8]. In addition, the SI covers two reviews [11,12].

Hong et al. investigated medullary thyroid cancer cells (MTC) [6]. The rationale was that mortalin inhibition suppressed human MTC cells in culture and in mouse xenografts and induced apoptosis and the downregulation of RET. Moreover, the agent MKT-077 inhibited mortalin and induced similar effects, but this compound is known to be toxic in animals. Different MKT-077 analogs (JG-98 and JG-194) inhibited propagation of TT and MZ-CRC-1 cells in 2D and 3D cultures. Both analogs also effectively suppressed the viability of TT and MZ-CRC-1 progenies resistant to vandetanib and cabozantinib. Moreover, JG-231 suppressed TT and MZ-CRC-1 xenografts in mice. These data suggest a potential of mortalin as a target for the design of a molecular therapy for MTC [6].

This SI also covered a study investigating human follicular TCC on the ISS in space [7]. A spaceflight induces various changes on the gene and protein expression level in human cells. Real microgravity (r-µg) altered many biological processes such as among others the cytoskeleton, extracellular matrix, focal adhesion, migration, proliferation, apoptosis, cell survival or growth behavior [13,14]. Using free-flow IEF to identify proteins under µg-conditions 235 proteins had been detected. 37 of this search appeared to be first identifications in human thyroid cells [15] and may serve as future targets. The CellBox-2 experiment focused on FTC-133 TCC flown to the ISS during the SpaceX CRS-13 cargo mission [7]. TCC grown on the ISS showed a similar secretory behavior as on Earth. Furthermore, the gene expression involved in processes such as proliferation, adhesion, growth and metastasis was suppressed in space [7]. In addition, a suppression of the NF-κB and ERK signaling pathway and an elevated angiopoietin 2 secretion after 10 d in orbit could be demonstrated [7]. These results are of clinical importance, but it has to be taken into account that the FTC-133 cell line is a stable platform for research on TC but does not fully represent the situation occurring in vivo.

Another study investigated *FOXE1*-induced transcriptional alterations in thyroid cells, not expressing endogenous *FOXE1* [9]. The authors demonstrated the *FOXE1*-dependent regulation of macrophage chemotaxis by thyroid cells in vitro and in vivo. This study detected a link between *FOXE1* and macrophages recruitment in the TCC microenvironment. The results indicate a function of this gene in the interaction of TCC and immune cells in relation to tumor development and progression [9].

Jiménez-Mora et al. demonstrated that the inhibition of ^V600E^BRAF induces a cytoprotective autophagy through AMPK in TCC [10]. The paper focused on the mechanisms whereby ^V600E^BRAF inhibition induced autophagy via activation of the LKB1-AMPK-ULK1 pathway. In addition, autophagy showed cell-protective properties and its blockage potentiates PLX4720-induced cell death of TCC carrying a ^V600E^BRAF mutation, both in vitro and in vivo. This data proposed new treatment strategies to target the AMPK pathway and/or autophagy that can contribute to enhance the efficacy of ^V600E^BRAF inhibitors and to overcome the acquired resistance to these compounds in TC [10].

A clinical study focused on the molecular mechanism and the clinical impact of ZNF677 expression in more than 1200 Middle Eastern papillary TC (PTC) and 15 metastatic tissues [8]. ZNF677 was frequently downregulated in primary PTC (13.6%, 168/1235) and the complete loss of ZNF677 expression was significantly associated with aggressive clinico-pathological markers such as extrathyroidal extension and distant metastases. Moreover, ZNF677 loss was an independent predictor of distant metastasis in PTC. In addition, the data showed that ZNF677 acts as a tumor suppressor, mediating its effect by inhibiting AKT phosphorylation. The authors demonstrated a key role for ZNF677 during carcinogenesis and metastasis formation in Middle Eastern PTC patients [8]. 

Finally, this SI also covered two reviews. The first review by Nguyen et al. summarized the current knowledge in clinicopathological and molecular features of secondary cancer (metastasis) to the thyroid and recent advances in the management of this finding [11]. This review provides clinicians with a comprehensive update regarding the clinical signs, diagnostic investigations, and current management of secondary cancer to the thyroid gland. Targeted therapies, such as multikinase inhibitors (MKIs) and immune checkpoint inhibitors might be effective in some of these patients.

The next review discussed the use of the MKIs lenvatinib, sorafenib and cabozantinib in RAI-refractory TC patients [12]. The authors reviewed the current knowledge of the MKIs in iodine-refractory DTC with focus on occurrence, mechanisms, and management of treatment-emergent hypertension (TE-HTN). The AE TE-HTN occurred during treatment with all compounds, but was well manageable as shown by the latest clinical trials. This was emphasized by the fact that lenvatinib was widely used as first-line drug of choice, despite its higher rate of TE-HTN [12].

Taken together, the seven excellent publications included in this SI demonstrate novel findings in the field of ‘*Recent Advances in Thryoid Cancer Research*’. The authors investigated several genes and molecular pathways which might serve as targets for future therapy in different TC types. They used cell culture and animal models or a large patient cohort to study molecular mechanisms to develop the rationale for a future targeted therapy.

I want to thank very much all the authors who contributed to this SI. I am convinced that the use of new molecular biological technologies together with a personalized medicine will increase the current knowledge of prevention, diagnosis and new therapies for TC. Moreover, novel approaches using OMICS technologies and Bioinformatics to search new proteins that may serve as new drug targets, will help to reduce the mortality of advanced metastatic TC types and secondary cancer to the thyroid.

## Data Availability

Not applicable.

## Abbreviations

AE	Adverse effect
AKT	Proteinkinase B/AKT serine/threonine kinase 1
AMPK	5′ adenosine monophosphate-activated protein kinase
BRAF	v-Raf murine sarcoma viral oncogene homolog B1
D	Day
ERK	Extracellular-signal regulated kinases
FOXE1	Forkhead box E1
FTC	Follicular thyroid cancer
HTN	Hypertension
ISS	International Space Station
LKB1	Liver kinase B1
MCS	Multicellular spheroids
MKIs	Multikinase inhibitors
MTC	Medullary thyroid cancer
mTOR	Mammalian target of rapamycin
PTC	Papillary thyroid cancer
r-µg	Real microgravity
SI	Special Issue
TC	Thyroid cancer
TCC	Thyroid cancer cells
TE-HTN	Treatment-emergent hypertension
2D	Two-dimensional
3D	Three-dimensional
ULK1	Unc-51 like autophagy activating kinase
ZNF677	Zinc finger protein 677
